# Novel insights into the unfolded protein response using *Pichia pastoris *specific DNA microarrays

**DOI:** 10.1186/1471-2164-9-390

**Published:** 2008-08-19

**Authors:** Alexandra Graf, Brigitte Gasser, Martin Dragosits, Michael Sauer, Germán G Leparc, Thomas Tüchler, David P Kreil, Diethard Mattanovich

**Affiliations:** 1 Institute of Applied Microbiology, Department of Biotechnology, University of Natural Resources and Applied Life Sciences Vienna, Muthgasse 18, 1190 Vienna, Austria; 2 School of Bioengineering, University of Applied Sciences FH Campus Vienna, Muthgasse 18, 1190 Vienna, Austria; 3 Vienna Science Chair of Bioinformatics, Department of Biotechnology, University of Natural Resources and Applied Life Sciences Vienna, Muthgasse 18, 1190 Vienna, Austria

## Abstract

**Background:**

DNA Microarrays are regarded as a valuable tool for basic and applied research in microbiology. However, for many industrially important microorganisms the lack of commercially available microarrays still hampers physiological research. Exemplarily, our understanding of protein folding and secretion in the yeast *Pichia pastoris *is presently widely dependent on conclusions drawn from analogies to *Saccharomyces cerevisiae*. To close this gap for a yeast species employed for its high capacity to produce heterologous proteins, we developed full genome DNA microarrays for *P. pastoris *and analyzed the unfolded protein response (UPR) in this yeast species, as compared to *S. cerevisiae*.

**Results:**

By combining the partially annotated gene list of *P. pastoris *with *de novo *gene finding a list of putative open reading frames was generated for which an oligonucleotide probe set was designed using the probe design tool TherMODO (a thermodynamic model-based oligoset design optimizer). To evaluate the performance of the novel array design, microarrays carrying the oligo set were hybridized with samples from treatments with dithiothreitol (DTT) or a strain overexpressing the UPR transcription factor *HAC1*, both compared with a wild type strain in normal medium as untreated control. DTT treatment was compared with literature data for *S. cerevisiae*, and revealed similarities, but also important differences between the two yeast species. Overexpression of *HAC1*, the most direct control for UPR genes, resulted in significant new understanding of this important regulatory pathway in *P. pastoris*, and generally in yeasts.

**Conclusion:**

The differences observed between *P. pastoris *and *S. cerevisiae *underline the importance of DNA microarrays for industrial production strains. *P. pastoris *reacts to DTT treatment mainly by the regulation of genes related to chemical stimulus, electron transport and respiration, while the overexpression of *HAC1 *induced many genes involved in translation, ribosome biogenesis, and organelle biosynthesis, indicating that the regulatory events triggered by DTT treatment only partially overlap with the reactions to overexpression of *HAC1*. The high reproducibility of the results achieved with two different oligo sets is a good indication for their robustness, and underlines the importance of less stringent selection of regulated features, in order to avoid a large number of false negative results.

## Background

Transcriptomics, the parallel quantification of many, or all transcripts of an organism in given conditions, has become a favorite tool for basic research [1]. Messenger-RNA regulation patterns of model organisms under many different conditions have become available during the last years. However, these methods are still not applicable for many industrially important organisms, mainly due to the lack of DNA microarrays targeting these organisms. A typical example is the yeast *Pichia pastoris*, which is widely applied for the production of recombinant proteins. Several approaches have been taken to derive transcriptomic data without specific microarrays. Sauer et al. [2] have applied heterologous hybridization of *P. pastoris *samples to *Saccharomyces cerevisiae *microarrays. Alternative methodological concepts like Transcript Analysis with the Aid of Affinity Capture (TRAC) [3] may be applied preferentially to subsets of the transcriptome [4], provided that genome sequence data are available. If this is not the case, total cDNA may be utilized as a source of probes, either by applying expressed sequence tags to microarrays [5] or employing RNA fingerprinting like cDNA-amplified fragment length polymorphism (cDNA-AFLP) [6], which has recently been applied to *Trichoderma reesei *[7]. These unannotated methods bear of course the disadvantage that specific hits may only be identified after sequencing their respective probes.

Therefore oligonucleotide microarrays have become the method of choice for many applications, although their design depends on the availability of a genomic sequence with good gene identification and annotation. The genome sequence of *P. pastoris *is not published yet. The data available from Integrated Genomics (IG, Chicago, IL, USA; [8]) contain a partial gene identification and annotation, so that additional effort in this direction was a first step necessary towards development of comprehensive DNA microarrays for this yeast species. There is a wide choice of computational gene finders available at the moment which can be classified into intrinsic and extrinsic prediction programs. Intrinsic or *de novo *gene finder only use information from the sequences to be studied, building statistical models to distinguish between coding and non-coding regions of the genome on the basis of biological sequence patterns [9-11]. Extrinsic gene finder utilize homology search to determine where protein coding regions are in the genome. Their applicability is therefore limited to organisms that have homologs in current databases that are correctly annotated. Because of this limitation it is common to integrate homology search with *de novo *prediction [12]. Most state of the art gene finders use a form of Hidden Markov Model (HMM) differing in the implementation and complexity of the model as well as the ease in which users can adapt the application to their needs [13].

It is well known that cross-hybridization can confound microarray results rendering good probe design an essential requirement for accurate microarray analyses. The specificity of oligonucleotides is determined by the Gibbs free energy (ΔG) of the hybridization reaction between potential binding partners. Highly specific probes will bind their target transcript much more strongly than any other transcript. Considering that microarray experiments are non-equilibrium measurements, it is desirable that microarray probes exhibit uniform thermodynamic properties, which many probe design tools aim to achieve by demanding a narrow distribution of the probe-target melting temperature T_m_. Ideally, probes should have a uniform binding free energy at the hybridization temperature *T*_*hyb *_[14].

Previous studies have demonstrated that industrial production strains may behave quite differently to laboratory strains and model organisms [15], which emphasizes the importance of analytical tools for industrially relevant strains and species. As an example, the unfolded protein response (UPR), a regulation circuit of high relevance for heterologous protein production in eukaryotic cells [16], has been shown to be differentially regulated in *P. pastoris *[4] compared to *S. cerevisiae *[17], which is the typical model species for hemiascomycete yeasts. The development of specific microarrays for *P. pastoris *was intended to allow a detailed analysis of UPR regulation in *P. pastoris*. As in previous transcriptomics work with *S. cerevisiae *the induction of UPR was either accomplished by addition of dithiothreitol (DTT) or tunicamycin, this work aimed at a comparison of DTT induced gene regulation in *P. pastoris *to that in *S. cerevisiae *published by Travers et al. [17]. Finally we aimed at the comparison of DTT induced regulation to the regulatory response to overexpression of *HAC1*, the transcription factor controlling the UPR. Transcriptional regulation of *HAC1 *overexpression has not been studied for yeasts so far, so that we expected valuable data to better define the core UPR regulated transcriptome.

## Results and Discussion

### Gene prediction and Oligo Design

To evaluate available gene finders for their performance on yeast genomes, three *de novo *gene finders (GeneMark, Glimmer3, GlimmerHMM) were tested on the genome sequence of *S. cerevisiae*. GeneMark and Glimmer3 work with a prokaryotic Hidden Markov Model (HMM) whereas GlimmerHMM employs a eukaryotic gene model. GeneMark was trained with coding and non-coding sequences of *S. cerevisiae*, building an HMM transition probability matrix of the 7^th ^order. Glimmer3 and GlimmerHMM could be trained directly on the genome in question without specifying coding and non-coding regions. In Lomsadze et al. [18] and Besemer and Borodovsky [9] the difficulty of eukaryotic gene finders in the prediction of genes for organisms with few introns is discussed and linked to a lack of data for representative exon – intron models. Our results confirmed that a gene finder written for eukaryotes (GlimmerHMM) could not be trained well on yeast genomes, introducing far too many introns into the predicted genes. Both prokaryotic versions performed much better, with GeneMark predicting less false negatives but more false positives than Glimmer3 (Table 1). Even though the positive prediction value was somewhat lower with GeneMark it was more important not to miss true positives than to achieve a lower rate of false positives. A further improvement could be achieved by a GeneMark model for lower eukaryotes, in which the prokaryotic algorithm is modified to use Kozak start sites instead of prokaryotic ribosomal binding sites. *P. pastoris *genes were predicted using this version of GeneMark with the lowest possible threshold (probability score t = 0.05) so that filter conditions could be better controlled at a later state. The prediction yielded a total of 26,471 putative genes for the genome of *P. pastoris*.

**Table 1:** Comparison of gene finder performance on yeast genomic sequence data

Gene finder	True positives	Partly	False positives	False negatives	Sensitivity (%)	Positive prediction value (%)
Glimmer3	75	13	21	31	73.9	68.8
GlimmerHMM	1	3	68(234)	115	3.2	1.4
GeneMark	81	16	32	22	81.5	62.7

Three different gene finders were tested on the genome sequence of *S. cerevisiae *chromosome 1 to evaluate the quality of gene prediction. Sensitivity = TP/(TP + FN), positive prediction value = TP/(TP + FP); For Glimmer HMM the column False Positives contains the number of genes and in brackets the number of exons.

In a WU-BLASTN search against *S. cerevisiae*, 6,374 sequences that were predicted by GeneMark, and 3,964 of the IG predictions produced hits with *S. cerevisiae *using an *E *value (Expectation value, [19]) of < 10^-4^, a hit length > 100 nucleotides and an identity of >50%. To reduce the redundancy within the data set the predicted genes were clustered into groups sharing more than 90% similarity using cd-hit [20]. From a total of 31,896 candidate sequences (GeneMark and IG predictions), 22,020 cd-hit groups were obtained. From the cluster file it was clear that some of the clusters had to be analyzed further before selecting target sequences for the oligo design. After the removal of all sequences that had a short length and a low prediction value, complex clusters were defined as clusters for which the minimum relative length of all sequences was smaller than 0.9. A total of 2,612 clusters fell into this category and were excluded at a first design stage.

Finally 19,508 predicted target sequences remained to be tested in the first microarray experiments. OligoArray 2.1 [21] was able to design oligonucleotide probes for 17,161 sequences ranging in length from 57 to 60 nucleotides.

### Validation arrays for the first list of predicted transcript sequences (Same-Same experiment)

With these probes 4 × 44 K slides were produced on the Agilent microarray platform and employed for an initial validation of the predicted transcript sequences by hybridization with the Pool samples of *P. pastoris *(for preparation of Pool samples see Material and Methods). One slide had to be discarded because of quality issues. For the remaining 12 arrays the number of probes showing a signal varied between 10,708 and 15,598. Of these, 7,980 had a signal on all 12 arrays, and only 951 probes showed no hybridization on all 12 arrays.

### Second, curated list of predicted target sequences and second oligo design

The results of the initial validation arrays were utilized to adapt the list of predicted genes, keeping all predictions for which a hybridization signal could be observed for all arrays plus all predictions with significant sequence similarity to annotated genes as well as all sequences with an average gene prediction score > 0.5. This approach allows for the fact that not all genes will have been actively expressed in the target samples. Additionally, predicted transcripts resulting from a subsequent analysis of the complex clusters were included at this stage. Of the 2,612 complex cluster that were not included in the design for the first batch of arrays, only 223 contained more than 2 sequences and for a further 14 no subsequence match of at least 60 nucleotides could be found within the last 1000 bases at the 3'-end. These 237 clusters were manually curated while the rest could be automatically reduced to one sequence. To make full use of the 15,208 features available on the Agilent microarray platform, it was decided to also include predicted sequences with somewhat lower gene prediction score that showed a hybridization signal in at least 8 of the 12 arrays. Finally, a selected set of 15,253 predicted transcript sequences was used as targets for probe design of a comprehensive *P. pastoris *microarray. While it is obvious that this list is larger than the expected number of open reading frames (6,000–7,000), as judged in comparison to other yeast species [22], we intentionally included more putative transcript sequences, as false positives with a distinct sequence will not negatively affect microarray design or experiments, in contrast to the damage of falsely excluding a potential transcript target.

Oligonucleotide probes were designed using a probe design tool developed in-house, a thermodynamic model-based oligoset optimizer ('TherMODO', [23]). TherMODO designed probes for 15,035 sequences, of which only 665 were predicted as having cross-hybridization potential. The TherMODO design was compared to probe design with eArray [24]. The distributions of ΔG and T_m _of both designs are shown in additional file 1. Clearly the TherMODO designed probes are more uniform in respect to the Gibbs free energy ΔG, indicating a superior hybridization performance [14].

**Figure 1:**
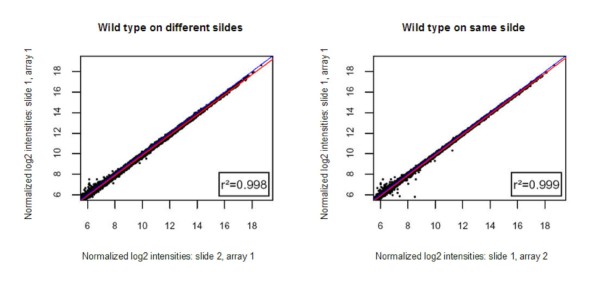
**Correlation of signal intensities**. Scatterplots of untreated wild type strain samples on (A) different arrays of the same slide; (B) different arrays on different slides. Red line: linear regression of the data; blue line: theoretical perfect correlation.

The final probe design was manufactured on 8 × 15 K slides by Agilent, and evaluated for reproducibility and biological meaningfulness. Pool samples were applied to 2 arrays on 2 slides each, including dye swap. The scatterplots show uniformly high correlations > 97% both within and between arrays, both on same and different slides, indicating high reproducibility of hybridization signals between identical samples. Exemplarily, a scatterplot of signal intensities derived from the same samples (wild type strain untreated) is shown in Figure 1. For the final gene list the annotation was improved in addition to the annotation provided by IG. This resulted in 3954 annotated ORFs, of which 2989 had an IG annotation. 965 newly annotated ORFs were found, and the annotation of 288 hypothetical proteins was confirmed. All annotated genes are listed in Additional file 2.

### Biological evaluation of the new microarrays

The performance of the new arrays was examined by a hybridization experiment using samples, for which transcript regulation data have been obtained before [4]. The biological question evaluated was the regulatory response of *P. pastoris *to constitutive overexpression of the active form of *S. cerevisiae HAC1*, the transcription factor controlling UPR target genes. By this approach, the regulation of 52 genes which have been studied before using TRAC [3] could be verified, with 80% of these genes showing the same regulation pattern for both methods (genes highlighted in bold in Additional file 2). This correlation is statistically significant based on calculating the regression (p = 8.8 · 10^-6^).

**Figure 2:**
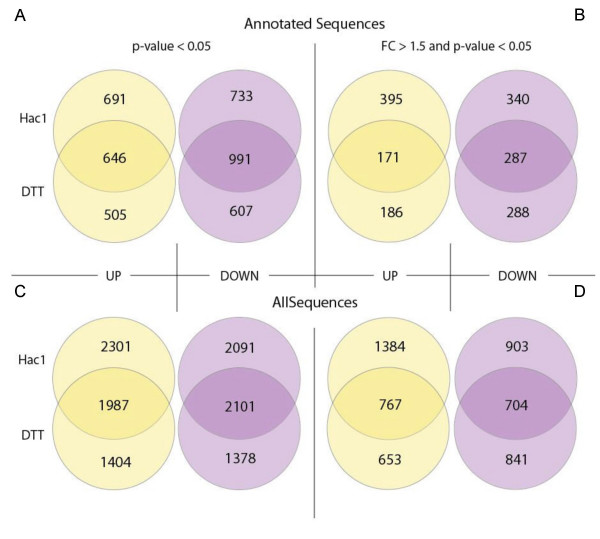
**Venn diagrams of differentially expressed genes upon DTT treatment or *HAC1* overexpression**. (A, B) Regulated hits with annotation; (C, D) all regulated features; (A, C) cut-off adjusted *p*-value < 0.05; (B, D) cut-off adjusted *p*-value < 0.05 and FC > 1.5.

**Figure 3:**
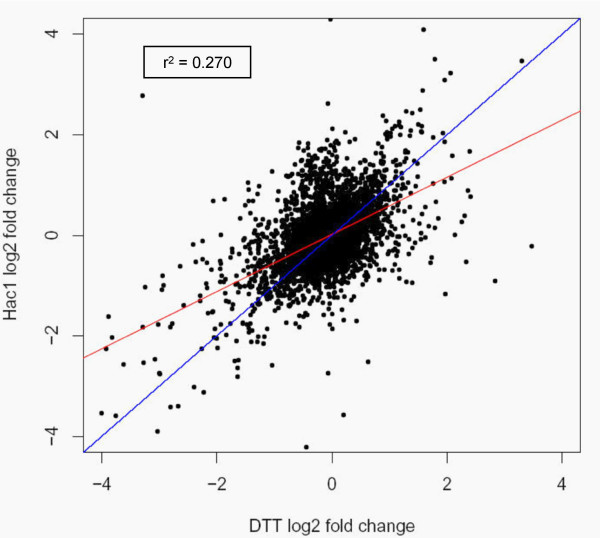
**Comparison of expression changes induced by DTT treatment and *HAC1* overexpression, respectively**. Log_2 _values of expression changes (log_2 _FC) caused by DTT (DTT treated wildtype *vs *untreated wildtype) and by Hac1 (*HAC1 *overexpression *vs *wildtype) are compared. The correlation coefficient r^2 ^is indicated. Red line: linear regression of the data; blue line: theoretical perfect correlation.

The similarities and differences of UPR induction and reaction to DTT stress have been discussed before [4,25,26]. To achieve further insight into this technologically relevant issue, we compared the gene regulation patterns of a *HAC1 *overexpressing strain *vs *wildtype control with the regulation pattern of the wildtype treated with DTT for 60 min *vs *the untreated control. Genes were qualified as significantly regulated with a *p*-value < 0.05 (adjusted for multiple testing). 11,262 of all features on the microarrays appeared as differentially regulated either upon DTT treatment or *HAC1 *overexpression, or both. 8,480 reacted to *HAC1*, and 6,870 to DTT, with an overlap of 4,088. Considering only the 3,954 annotated genes, a similar pattern is observed with roughly half of the regulated genes overlapping between DTT and *HAC1*, and another half being typical only for either of the treatments (Figure 2). Accordingly, the correlation of log fold changes of the two treatments is apparent but rather weak (Figure 3). While DTT treatment is widely accepted as a standard inducer of UPR, these observations indicate that the gene regulation pattern triggered by the UPR transcription factor Hac1 differs to a significant extent from that exerted by DTT.

**Figure 4:**
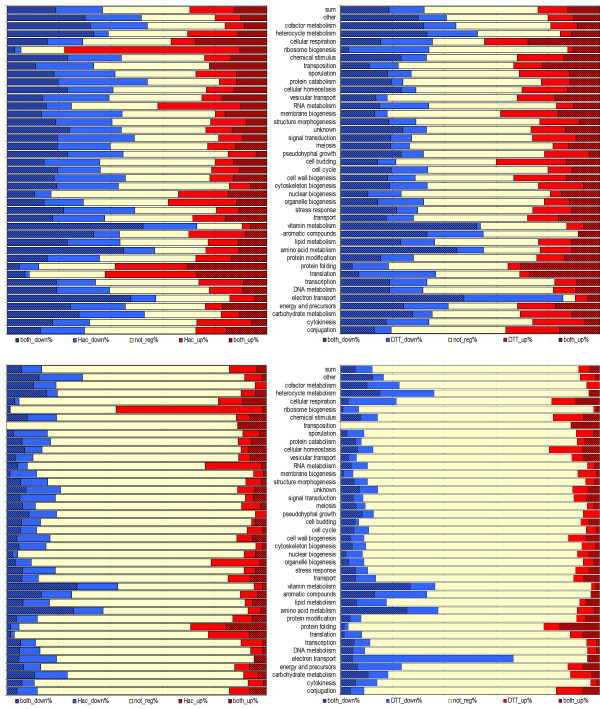
**Fractions of up- and downregulated genes in functional groups**. Relative numbers of upregulated (red), downregulated (blue) and unregulated (yellow) genes categorized in GO biological process terms upon *HAC1 *overexpression (left panel) and DTT treatment (right panel). Shaded in black: regulated in both treatments. Upper panels: cut-off *p*-value < 0.05, lower panel cut-off *p*-value < 0.05 and FC > 1.5. The results of significance testing (Fisher's exact test) are given in additional file 4.

As previous research on transcriptome regulation upon UPR induction usually employs a fold change (FC) cut-off to highlight the strongly regulated genes, we decided to introduce FC > 1.5 as a second criterion to identify more strongly regulated genes for further detailed analysis (Volcano plots visualizing the two criteria are provided in Additional file 3). Although the introduction of a FC cut-off alters the absolute number of regulated genes, it does not alter the relative distribution of regulated genes categorized into functional groups (GO slim biological process), as can be seen in Figure 4 and Additional file 4.

### Comparison of UPR induction by DTT in *P. pastoris *and *S. cerevisiae*

In order to compare the effects of DTT treatment in *S. cerevisiae *with those in *P. pastoris*, the data published by Travers et al. [17] for 60 min treatment of *S. cerevisiae *with DTT were evaluated alongside with our results for *P. pastoris*. All genes of *S. cerevisiae *which were listed in [17] and for which homologs in *P. pastoris *were identified were classified as upregulated, downregulated or unregulated. In order to compare the two data sets, a cutoff of 1.5 fold differential expression was set in both to define regulated genes. A significance threshold on *p*-values could not be employed, as these data were not provided for *S. cerevisiae*. 48% of these genes defined as regulated or unregulated reacted in *P. pastoris *just as in *S. cerevisiae*.

A closer evaluation revealed that certain GO groups were regulated very similarly in both yeast species, while others showed only a low degree of similarity (Table 2). Fisher's exact test was performed to evaluate the significance of groups with low similarity. Especially the GO groups 'translocation', 'protein folding', 'protein degradation', and to some extent 'glycosylation' and 'transport' showed high degrees of similarity. In some GO groups, only some subgroups reacted similarly while others behaved differently in the two yeasts. Of the 'glycosylation' group, core oligosaccharide synthesis and glycosyltransferase genes behaved very similarly, while glycoprotein processing, GPI anchoring and O-glycosylation related genes were regulated significantly different (p < 0.05). In the 'protein degradation' group, more similarity was observed for ERAD genes than for ubiquitin/proteasome related genes. Among the 'transport' gene group, budding, fusion and retrieval of ER to Golgi showed a high degree of similar regulation, contrary to the subgroup distal secretion. Low similarities were observed for 'lipid metabolism', 'vacuolar protein sorting' and 'cell wall biogenesis' genes. It becomes obvious that core UPR genes related to protein translocation, folding and ER transport, as well as core N-glycosylation react similarly to DTT treatment in *P. pastoris *as compared to *S. cerevisiae*, while genes involved in processes which are more distal from ER protein folding behave more differently, indicating that those processes (like functions in the Golgi, [27]) differ significantly between the two yeasts.

**Table 2:** Similarity of gene regulation between *P. pastoris* and *S. cerevisiae* upon DTT treatment

Function	Subfunction	No. of similarly regulated/total	% similar regulation
Translocation	total	4/6	67
Glycosylation	total	11/22	50
	Core oligosaccharide synthesis	3/4	75
	Oligosaccharyltransferase	4/4	100
	Glycoprotein processing	1/5	20
	GPI anchoring	1/4	25
	Golgi/O-linked	2/5	40
Protein Folding	total	5/8	63
	Chaperones	3/5	60
	Disulfide bond formation	2/3	67
Protein Degradation	total	4/5	80
	ERAD	3/3	100
	Ubiquitin/Proteasome	1/2	50
Transport	total	11/20	55
	Budding (ER-Golgi)	4/7	57
	Fusion (ER-Golgi)	1/1	100
	Retrieval (ER-Golgi)	4/5	80
	Distal secretion	2/7	29
Lipid Metabolism	total	5/18	28
	Fatty acid metabolism	0/4	0
	Heme biosynthesis	2/5	40
	Phospholipid biosynthesis	2/6	33
	Sphingolipid biosynthesis	0/1	0
	Sterol metabolism	1/2	50
Vacuolar Protein Sorting	total	1/4	25
Cell Wall Biogenesis	total	4/10	40

All genes that were indicated in [17] as core UPR genes in *S. cerevisiae *and having an annotation in *P. pastoris *were grouped by their GO process functions. Similar regulation of a gene means upregulated, downregulated or below cut-off, respectively, in both yeasts.

### Overexpression of Hac1 triggers a different regulation pattern compared to DTT treatment

In most previous studies of the UPR in lower eukaryotic cells, treatment with DTT or tunicamycin, or heterologous protein expression has been employed to trigger the UPR. This study clearly indicates that the set of regulatory events triggered by DTT analysis only partially overlaps with the reactions to constitutive expression of the activated form of the UPR transcription factor Hac1 (see Figures 2 and 3). Interestingly, both treatments resulted in the same amount of genes being down-regulated as being up-regulated, a fact that has been neglected to some extent in the existing literature.

Those genes appearing beyond the threshold (*p*-value < 0.05 and FC >1.5) were subjected to a more detailed comparison between the effects of DTT treatment and Hac1 induced regulation. The relative numbers of up- and downregulated genes in each GO biological process term based on the SGD GO slim tool [28] are depicted in Figure 4.

A pattern common to both treatments is the down-regulation of major metabolic processes like carbohydrate, amino acid and lipid metabolism, as well as that of vitamins, cofactors and aromatic and heterocyclic compounds. This makes it obvious that the UPR has a major impact on decreasing both catabolic and anabolic processes. On the other side, both treatments lead to up-regulation of protein folding and vesicular transport. These effects are in line with the published literature, indicating the cellular reaction towards alleviation of the UPR [4,25,26,17].

As expected, the genes coding for classical UPR targets are induced both in Hac1 overproducing and in DTT stressed cells, and genes underlined in the following paragraphs have been identified as UPR targets in previous studies. Especially the ER folding catalysts *PDI1* and *ERO1*, the DnaJ homologs *JEM1* and *SCJ1*, the ER resident chaperones *CNE1 *(calnexin), *KAR2*/BiP and *LHS1* and the mitochondrial chaperones *HSP60 *and *SSC1 *are significantly up-regulated in both conditions. Among the functional group of 'protein modification' the majority of up-regulated genes belong to the core oligosaccharide synthesis (*DPM1*, *DIE2*), oligosaccharyltransferase complex (*OST1*, *OST2*, *OST3*, *SWP1*, *STT3*, *WBP1*), glycoprotein processing (*ALG2, ALG7*, *SEC53*), GPI anchor biosynthesis (*GPI2*, *GPI14, PSA1*) and Golgi/O-linked glycosylation (*PMT1*, *PMT2*, *PMT4, PMT6*). Besides these, several genes coding for the translocon pore complex (*SEC61*, *SEC62*, *SEC63*, *SEC72*, *SSS1*), which aid the translocation of nascent polypeptides into the ER, are induced. Higashio and Kohno [29] describe the stimulation of ER-to-Golgi transport through the UPR by inducing COPII vesicle formation. In this context, we see *SEC23*, *SEC24*, *SFB2*, *YIP3*, and *ERV2 *upregulated. However, also proteins building the COPI coatomer, which are required for retrograde Golgi-to-ER transport, show increased transcription levels upon ER stress in our experiments (*COP1*, *RET2*, *SEC21*, *SEC27*).

While we cannot give any information on ERAD regulation, as *HRD1* is the only annotated gene of this protein degradation process (up-regulated in the Hac1 strain), we observed the down-regulation of some components involved in the assembly of the 20 S core of the 26 S proteasome (*ADD66*, *PRE1*, *PRE4*, *SCL1*) and ubiquitin *UBI4 *upon constitutive UPR activation. In this context, Shaffer et al. [30] describe reduced degradation of newly synthesized proteins in XBP1-overexpressing human Raji cells.

Induction of genes encoding cytosolic chaperones (Cns1, Jjj3, Hsp82, Ssa1, Ssa2, Sse1, Ydj1, Zuo1) can only be seen in the Hac1-overproducing strain. Additionally, the ER-resident Pdi homolog Mpd1 and two members of the PPIases (*FPR4 *and *CPR6*) are only up-regulated in the engineered strain, but not upon DTT addition.

One of the most striking patterns is the significant up-regulation of a large number of genes with functions in ribosomal biogenesis (233 genes assigned to the GO-categories 'ribosome biogenesis and assembly' and 'RNA metabolic process'). Most of these genes are contributing to rRNA processing (RRP family) and ribosome subunit nuclear export and assembly, while the ribosomal proteins (RPS and RPL families) themselves are not among the regulated genes for *P. pastoris *(see Additional file 2). No genes with a function in mRNA decay show increased transcription levels. The induction of the above functional categories came as a surprise, as translational down-regulation of proteins involved in ribosomal biogenesis was recently reported when *S. cerevisiae *cell were treated with DTT [31]. In contrast, the transcription levels of 9 out of the 16 mRNAs listed by these authors are enhanced in our study. Transcriptional down-regulation of ribosomal proteins during ER stress conditions was also revealed when reanalysing the raw data provided by Travers et al. [17]. However, Shaffer et al. [30] describe an increase in total protein synthesis as well as in the number of assembled ribosomes upon the overexpression of the mammalian Hac1 homolog XBP1 in Raji cells, but did not observe upregulation of genes related to ribosome biogenesis. A similar effect was observed after XBP1 overexpression in CHO-K1 cells [32]. These results may be an indication that the positive effect of overexpression of the UPR transcription factor on heterologous protein production [33,16,35] results not just from stimulation of folding and secretion of proteins but also their synthesis. The induction of protein folding related genes upon Hac1 overexpression is in line with the literature on UPR effects, while an impact on organelle biosynthesis other than ER and Golgi has so far only been described for mammalian cells.

The stimulatory effects of XBP1 induction on ribosomes and organelle synthesis in mammalian cells like lymphocytes have been attributed to their function as dedicated protein factories. On the other hand the UPR in lower eukaryotes should rather serve to alleviate the load of unfolded, aggregation prone protein. It will be of interest in the future to investigate whether Hac1 stimulates ribosome biogenesis in other yeasts and fungi as well, and whether this leads to increased translation.

In this context, it is worthwhile to mention the induction of two pathways leading to the unusual post-translationally modified amino acid derivatives diphthamide and hypusine which are exclusively found in eukaryotic translation elongation factors 2 (eEF2) and 5 (eEF5), respectively [36,37]. As these biosynthetic pathways are rather complex, and outstanding in the otherwise downregulated group of 'amino acid biosynthesis', this induction underlines the increased demand for protein synthesis.

Furthermore, we observe that ER stress leads to increased transcription of genes coding for the large and small subunits of the mitochondrial ribosomes (*MRPS*, *RSM *and *MRPL *families), mitochondrial translation initiation and elongation factors (*IFM1*, *MEF1*, *MEF2*) and mitochondrial DNA polymerase (*MIP1*). Several essential constituents of the mitochondrial inner membrane presequence translocase (*TIM *family) are also up-regulated, indicating increased necessity for protein import into the mitochondria. Similarly, XBP1 was shown to increase mitochondrial mass and function in two types of mammalian cells [30].

While previous studies analysing UPR regulation mainly focus on up-regulated genes [17], more than half of the genes identified in our study to be regulated are strongly down-regulated (at least 1.5 fold). As can be seen in Figure 4, anabolic processes such as vitamin production, amino acid and aromatic compound biosynthesis, heterocycle metabolic processes, carbohydrate, lipid and cofactor metabolism are among the most prominent repressed classes in both DTT-treated as well as Hac1-overproducing cells. The down-regulation of energy consuming biosynthetic pathways emerges as a general picture during ER stress conditions. However, it becomes obvious that the response to the folding perturbation agent DTT strongly differs from constitutive UPR induction by Hac1-overproduction. Especially the prominent down-regulation of genes belonging to 'electron transport' and 'cellular respiration' can easily be explained by the strong reducing capacities of DTT. Prominent members of the mitochondrial inner membrane electron transport chain such as subunits of the cytochrome c oxidase (*COX4, COX4, COX5A, COX13*) and the ubiquinol cytochrome-c reductase complex (*COR1, QRC6, QRC7, QRC9, RIP1*) are significantly repressed upon DTT treatment. Additionally, cytochrome c (*CYC1*), cytochrome c1 (*CYT1*) and cytochrome c heme lyase (*CYC3*) are only under DTT-dependent repression (GO: 'generation of precursor metabolites and energy'). The reducing features of DTT are most probably also the reason for the up-regulation of genes involved in the upkeeping of 'cellular homeostasis' and clearly, addition of DTT is provoking a 'response to a chemical stimulus'.

Down-regulated genes appearing in both Hac1 and DTT in the 'protein modification' group focus on protein kinases (*CDC5, CDH1, DBF2*) and components of the ubiquitinylation complex (*BUL1, CUL3*) involved in cell cycle regulation driving the cells towards mitotic exit (*CDC5, CDH1*, *MOB1*). These effects are even more pronounced in the Hac1-strain, where several more histone modifying enzymes as well as cycline-dependent protein kinases and components of the protein kinase C signalling pathway show reduced transcription levels compared to the wild type. Unlike reported for the filamentous fungi *T. reesei *[7] and *A. nidulans *[26], genes encoding the histones H2A, H2B, H3 and H4 appear to be down-regulated upon secretion stress in *P. pastoris*.

No clear picture emerges regarding the regulation of 'lipid metabolism': While sterol and ergosterol biosynthesis tend to be inhibited, the production of sphingolipid precursor substances is enhanced. On the other hand, a down-regulation of the major cell wall constituents (β-1,3 glucanases *BGL2 *and *EXG1*, cell wall mannoproteins *CCW12*, *CWP2 *and *TPI1*, GPI-glycoproteins *GAS1 *and *SED1*, *PST1*) and genes coding for proteins required for the transport of cell wall components to the cell surface (*SBE22*) is manifest. Taken together, these results indicate a significant remodelling process regarding the *P. pastoris *cell envelope during ER stress conditions.

Interestingly, the major groups of metabolic genes were down-regulated upon Hac1 overexpression, indicating a decrease of the supply of metabolites. However, it should be noted that no reduction of the specific growth rate was observed as compared to the wild type strain (μ = 0.37 and 0.39 h^-1^, respectively). A reduction of metabolic processes, and amino acid synthesis in particular, is contradictory to translation stimulation. Further research will be needed to elucidate the overall regulatory pattern of UPR in respect to protein synthesis.

## Conclusion

Additional gene finding and annotation added to the available data for *P. pastoris *lead to a list of approximately 4,000 genes with a putative identification of their function, and 11,000 more potential open reading frames. An oligonucleotide probe set was designed, the hybridization results were evaluated for reproducibility, and results from a biologically relevant analysis were tested for meaningfulness. In a direct comparison to *S. cerevisiae *employing DTT treatment for UPR induction, 45 out of 93 genes reacted similarly. The differences thus observed between *P. pastoris *and *S. cerevisiae *underline the importance of DNA microarrays for industrial production strains. *HAC1 *overexpression in *P. pastoris *obviously leads to induction of many genes involved in translation: most genes of ribosome biogenesis, as well as many related to RNA metabolism and translation were up-regulated, an effect that has never been observed in yeasts and filamentous fungi so far.

The upregulation of ribosomal biogenesis, RNA metabolism, translation, and organelle biosynthesis is specific for *HAC1 *overexpression and not observed with DTT treatment, while the latter leads specifically to the upregulation of genes related to chemical stimulus, and the downregulation in the groups electron transport and respiration, so that these reactions have to be regarded as specific for the treatment with a reducing agent rather than UPR regulated.

## Methods

### Gene Prediction and Sequence Selection

Gene prediction and the selection of sequences for oligonucleotide probes were based on sequenced contigs of the *P. pastoris *genome including predictions of protein coding genes, available through Integrated Genomics [8]. The number of predicted genes was 5,425 of which 3,680 had an assigned function. The ORFs were made up of experimentally identified genes, as well as ORFs predicted by a proprietary gene finder [38].

To validate and possibly improve these predictions, *de novo *gene finding was conducted. First three *de novo *gene finder (GeneMark, Glimmer3, GlimmerHMM) were tested on the genome sequence of *S. cerevisiae *(data from BioMart, [39]) to evaluate their performance on yeast genomes. As described in Results and Discussion, GeneMark [40] was selected for further gene prediction on the *P. pastoris *genome sequence. To run the gene prediction it was necessary to train GeneMark on *S. cerevisiae *by building a matrix with transition probabilities for coding and non-coding regions used by the Hidden Markov Model (HMM) of the program. With the amount of data available we were able to generate a matrix of the 7^th ^order. The genes of *P. pastoris *were predicted using the *S. cerevisiae *matrix and the lowest possible probability score cut-off (t = 0.05). In the initial stage of the microarray design the aim was to predict as many putative ORFs as possible. In this context a higher false positives rate was accepted in order to keep the false negatives rate as low as possible.

The predicted sequences were merged with data from IG and clustered by running cd-hit [20] with a similarity cut-off of 90%. For all of the resulting sequences a BLASTX search was done against *S. cerevisiae *using WU-BLAST [19]. Blast data was further filtered for length (cutoff 55 bp) and low prediction score. Clusters comprised of more than one gene were represented by the longest sequence, or curated manually, if appropriate.

From this first gene list (PpaV1) microarrays were analyzed as described below. Spots with a positive signal were determined using the mean plus one standard deviation of the negative control probes as a cut-off. Sequences were selected if they were positive in at least 8 out of 12 arrays. This criterion was chosen to fill the array capacity. Additionally all sequences with a probability score higher than 0.5 or having an annotation were kept for the second set of sequences (PpaV2).

### Annotation

For the PpaV2 sequence set the program cd-hit-est [20] was used to find all ORFs that had a global identity of > 80% with *S. cerevisiae*. WU-BLASTX and WU-TBLASTN searches were conducted against *S. cerevisiae*, using a low complexity filter and E < 10^-7^. For all the sequences that did not have a match with *S. cerevisiae *under these conditions the two BLAST searches were repeated against the SwissProt/TrEMBL [41] database. A perl script was developed to summarize and compare the BLAST results.

### Oligo Design and Array platform

Oligos for the PpaV1 sequences were designed with the Program OligoArray 2.1 [21] to match the melting temperature distribution of Agilent's *S. cerevisiae *oligos on the Yeast Oligo Microarray (V2), design number 013384.

The oligo-set for the PpaV2 sequence set was designed using the thermodynamic model-based oligoset optimizer 'TherMODO'. This tool incorporates advanced quantitative models for probe-target binding region accessibility and position-dependent target labelling efficiency, and replaces the common greedy search algorithm by a global set optimization step, achieving high discrimination power for particularly uniform probe sets [23]. Probes for Agilent arrays are limited to a maximum length of 60 nucleotides by the manufacturing process. For increased flexibility in the probe design, the oligoset design optimization considered probes ranging in length from 57 to 60 nucleotides.

These arrays were produced on Agilent 60 mer oligonucleotide high density arrays 4 × 44 K (with 42,034 available features) for PpaV1 and 8 × 15 K (with 15,208 available features) for PpaV2.

### Experimental Design

For the first batch of arrays a same-same design was used, employing six replicates each of Pool 1 and of Pool 2. The aim of this experiment was to determine which of the probes hybridize to *P. pastoris *targets. For the second batch of arrays a two-state comparison set up was chosen with 6 replicates for each experiment of which 3 were dye swapped.

### Strains und Cultures

For the first batch of arrays the aim was to determine which of the predicted probes hybridize with targets from *P. pastoris*. To make sure that many genes were active it was important to pool samples from various conditions of the cells. Samples were taken from two different *P. pastoris *strains, X-33 and CBS2612, grown on different media and taken at both exponential and stationary growth phase. The media were YP Medium (1% yeast extract, 2% peptone and either 2% glucose, 2% glycerol or 0.5% methanol as carbon source), Buffered Minimal Medium (1.34% yeast nitrogen base, 4 × 10^-5^% biotin, 100 mM potassium phosphate pH 6.0 and either 2% glucose, 2% glycerol or 0.5% methanol as carbon source), and Buffered Minimal Medium described above supplemented with amino acids (0.005% of L-glutamic acid, L-methionine, L-lysine, L-leucine and L-isoleucine). The samples were combined into two pools with Pool 1 containing 18 samples from the exponential growth phase and Pool 2 containing 18 samples from the stationary phase. Both pools additionally contained seven chemostat samples of the strain X-33 3H6Fab, grown as in [42].

For the UPR experiments, strains GS115 HAC1, constitutively overproducing the activated form of *S. cerevisiae *Hac1, as described in Gasser et al. [33,4], as well as GS115 transformed with the empty vector pGAPHIS (a histidine prototrophic isogenic strain of GS115) were cultivated in YPD (YP as above with glucose) at 28°C. After growing the cultures to an OD_600 _= 5.7, dithiothreitol (2.5 mM) was added where appropriate. After 1 more hour of cultivation, 1 ml culture was added to 0.5 ml precooled phenol solution (5% in absolute ethanol) and centrifuged immediately for 30 sec at 13.000 rpm. After discarding the supernatants the pellets were frozen at -80°C.

### RNA Isolation

All samples were resuspended with 1 mL TRI Reagent (Sigma). Cells were disrupted after addition of 500 μL glass-beads with a Thermo Savant Fastprep FP120 Ribolyzer by treatments of 2 × 20 sec at 6.5 ms^-1^. RNA was extracted with chloroform, precipitated with isopropanol, washed with 75% ethanol and dissolved with diethylpyrocarbonate treated water. The extracted RNAs were quantified via absorption at 260 and 280 nm. The quality of the RNA samples was verified with the Agilent Bioanalyzer 2100 and RNA 6000 Nano Assay kit (Agilent Technologies, California).

### Labeling and Hybridization

Hybridization targets for *P. pastoris *microarrays were prepared according to Agilent's Two-Color Microarray-Based Gene Expression Analysis protocol (Version 5.5, February 2007). Purification of the labelled and amplified RNA was conducted using RNeasy mini spin columns (Qiagen). The quality of labelled cRNA was evaluated on the Agilent Bioanalyzer 2100 and quantified using a ND-1000 NanoDrop Spectrophotometer. Fragmented cRNA samples were applied to the individual arrays. The slides were placed into Agilent hybridization oven and hybridized for 17 h, at 65°C and 10 rpm.

### Microarray Analysis

Slides were scanned with an Agilent MicroArray Scanner and intensities were extracted using Agilent's Feature Extraction software (version 9.1). The resulting data was imported into R where data pre-processing and normalization was performed. In the pre-processing step all outliers and saturated spots were given the weight zero. After plotting the data we decided to refrain from background correction since it has the tendency to add more noise to the data [43]. The data were normalized using locally weighted MA-scatterplot smoothing (LOESS) followed by a between array scale normalization. Both functions are available within the limma package of R [44]. For the selection of differentially expressed genes linear models were fitted to the log-ratios of the expression data separately for each gene. An empirical Bayes approach was used to shrink the probe-wise sample variances towards a common value yielding a moderated *t*-statistic per gene [45]. *P*-values were corrected for multiple testing using Holm's method [46]. Features were defined as differentially expressed if they had a *p*-value < 0.05. For the identification of stronger regulatory effects an additional cut-off for the fold change (FC) of 1.5 > FC > 1/1.5 was applied. Description of the platform, array, raw data as well as processed data were deposited at ArrayExpress [47] under the accession numbers A-MEXP-1157.

All annotated *P. pastoris *genes were categorized into GO biological process terms using the SGD GO slim tool [28], whereby *P. pastoris *specific genes were included into the term 'other'. The significance of a deviation of the number of up- or downregulated genes in each group from the average was verified with a Fisher test (Additional file 4).

## Authors' contributions

AG performed gene finding and annotation, statistical data analysis, supported data evaluation, and drafted part of the manuscript. BG performed data evaluation, supported annotation, study design, array design and drafted part of the manuscript. MD performed the cultivations and hybridizations. MS contributed to study design, annotation and array design. GGL developed the employed probe design tool and supported the array design. TT developed the quantitative model for the position-dependent target labelling efficiency and adapted it for the relevant end-primed labelling protocol. DPK supervised gene identification and annotation, supervised and contributed to the development of the employed probe design tool, and contributed to the manuscript. DM conceived of the study, and participated in data evaluation and manuscript drafting. All authors read and approved the final manuscript.

## Supplementary Material

Additional File 1 **Thermodynamic properties of the TherMODO probe design compared to probes designed through Agilent's eArray**. Distribution of Gibbs free energy ΔG (A) and the probe-target melting temperature T_m _(B) of the oligo sets. The upper row (1) shows the oligos designed through eArray and the lower row (2) the oligos designed with TherMODO. PpaV2 is the name of the second set of sequences as described in the Materials and Methods section. Click here for file

Additional File 2 **Differential expression values of all annotated genes upon DTT treatment and *HAC1 *overexpression**. Differential expression values and adjusted *p*-values of all annotated genes of *P. pastoris*, denominated with the gene name of their respective *S. cerevisiae *homolog. Genes that were tested with TRAC as a different method for transcript quantification are highlighted in bold letters. Legend of headers: id – internal unique identifier of sequence; sequ_id – ERGO identifier (RPPA.) or gene finder identifier (orf.) respectively; DTT_logFC – log_2_ fold change of DTT treatment compared to control; *HAC1 * logFC – log_2_ fold change of *HAC1 * overexpression compared to control; Gene name – Standard gene name or if missing systematic ORF name according to *S. cerevisiae *nomenclature; GO – Gene Ontology term (for descriptions see additional file 4). If a gene is present in a certain GO group it has a 1 in the respective column, if not it has a 0. Click here for file

Additional File 3 **Volcano plots of fold change vs. adjusted *p*-values**. (A) DTT treatment; (B) *HAC1 *overexpression. Blue line: *p*-value cut-off *p *> 0.05; red lines: optional fold change cut-off FC > 1.5. Click here for file

Additional File 4 **Fisher's exact test of the up/down regulated gene groups upon DTT treatment and *HAC1 *overexpression**. Fisher's exact test was applied to test significance of the up- and downregulated gene groups displayed in figure 4. *p*_*adj *_values are given for each GO group. Legend of headers: group – Gene Ontology term; Description – Gene Ontology description; odds.ratio – measure of independence between variables; adj.p – Holm adjusted *p*-value; *HAC1 * up/down – up/down regulated in *HAC1 * overexpression experiment; DTT up/down – up/down regulated in DTT experiment. The first work sheet represents results using only a *p*-value cut-off *p *> 0.05, the second work sheet represents results using a *p*-value cut-off *p *> 0.05 and a fold change cut-off FC > 1.5. Click here for file
