# Supplementary figures and images for: Novel insights into the unfolded protein response using Pichia pastoris specific DNA microarrays
Source: BMC Genomics. 2008 Aug 19;9:390. doi: 10.1186/1471-2164-9-390 (PMC2533675; doi:10.1186/1471-2164-9-390)

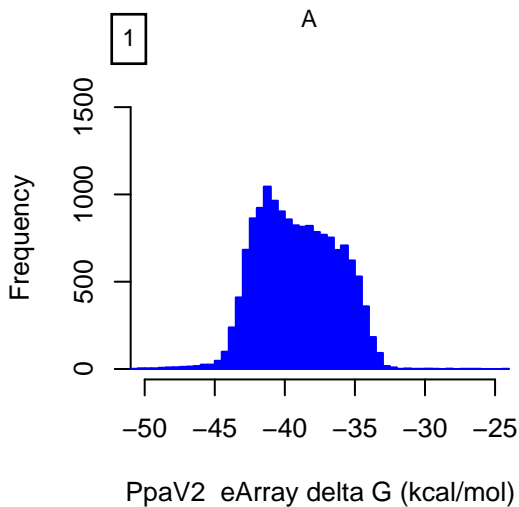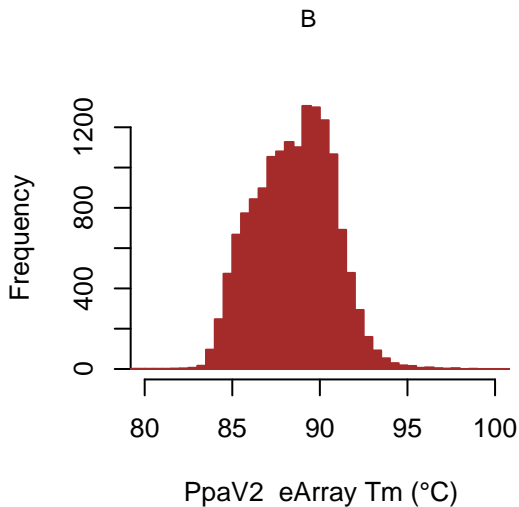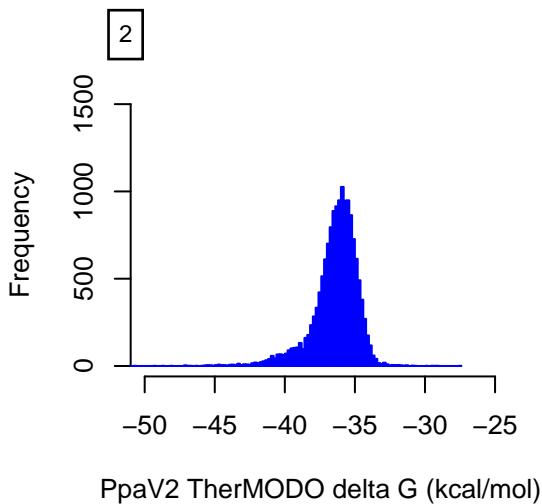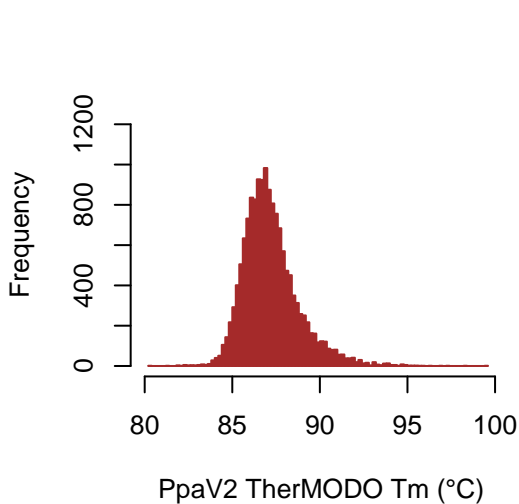

Supplement: Additional File 1 — Thermodynamic properties of the TherMODO probe design compared to probes designed through Agilent's eArray. Distribution of Gibbs free energy ΔG (A) and the probe-target melting temperature Tm (B) of the oligo sets. The upper row (1) shows the oligos designed through eArray and the lower row (2) the oligos designed with TherMODO. PpaV2 is the name of the second set of sequences as described in the Materials and Methods section. [file 1471-2164-9-390-S1.pdf]

DTT

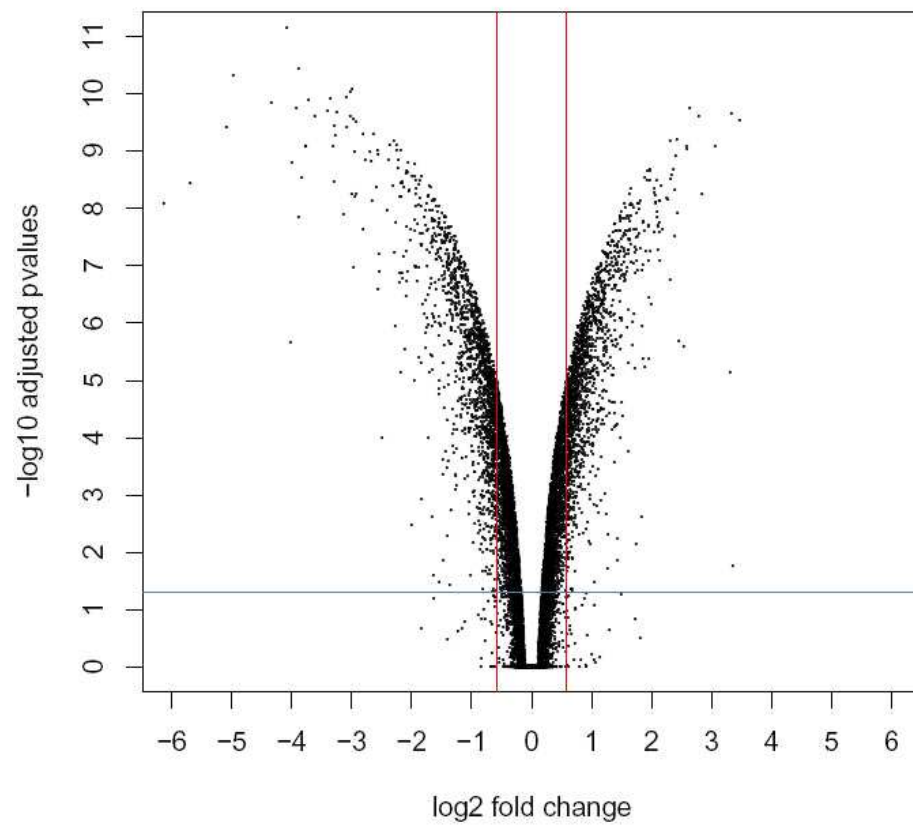

Hac1

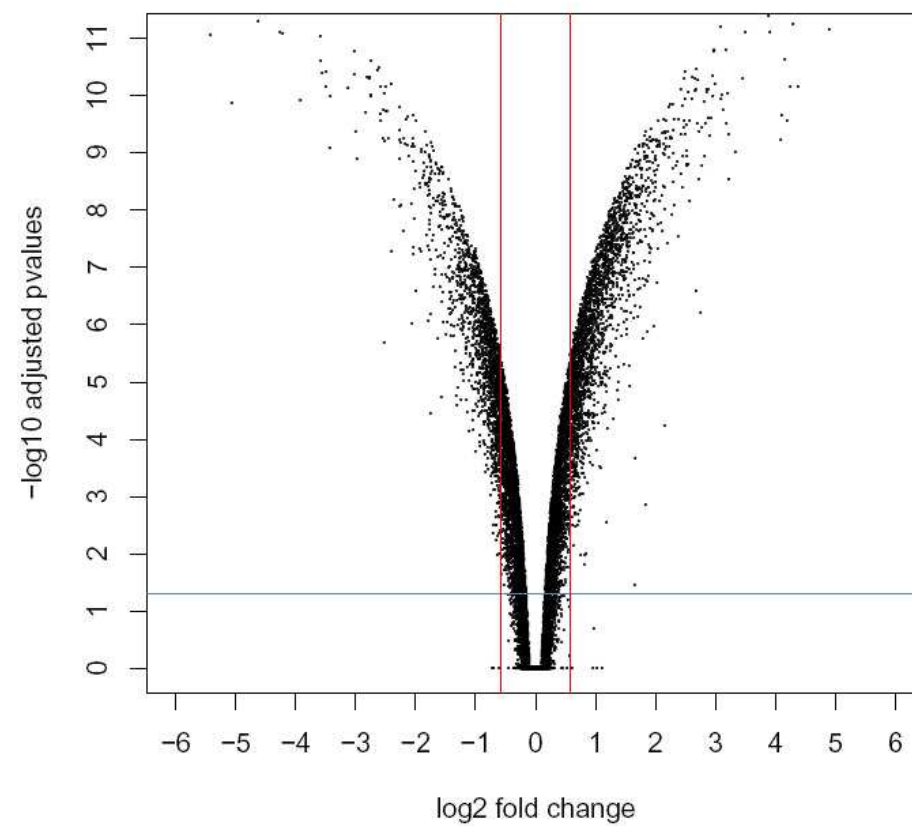

Supplement: Additional File 3 — Volcano plots of fold change vs. adjusted p-values. (A) DTT treatment; (B) HAC1 overexpression. Blue line: p-value cut-off p > 0.05; red lines: optional fold change cut-off FC > 1.5. [file 1471-2164-9-390-S3.pdf]
